# Updates in novel immunotherapeutic strategies for relapsed/refractory AML

**DOI:** 10.3389/fonc.2024.1374963

**Published:** 2024-12-04

**Authors:** Sawyer Bawek, Sayuri Gurusinghe, Matthew Burwinkel, Amanda Przespolewski

**Affiliations:** ^1^ Department of Internal Medicine, University at Buffalo, Buffalo, NY, United States; ^2^ Leukemia Service, Department of Medicine, Roswell Park Comprehensive Cancer Center, Buffalo, NY, United States; ^3^ Cell Therapy, Bristol Myers Squibb, Princeton, NJ, United States

**Keywords:** acute myeloid leukemia, immunotherapy, relapsed/refractory, cellular therapy, CAR-T

## Abstract

Acute myeloid leukemia (AML) is a severe hematological malignancy with poor outcomes, particularly in older adults. Traditional treatment options like high-dose chemotherapy often lead to refractory or relapsed AML, with even worse outcomes. New therapies for relapsed and refractory AML are needed, and this review explores the most recent advancements in immunotherapy in AML. Checkpoint Inhibitors utilizing innate or adaptive immune targeting have shown potential to improve AML outcomes when combined with hypomethylating agents and chemotherapy. The use of adoptive cell therapy in AML demonstrates promising early data, however, there is a need for better target selection. Although early in development, both vaccine therapy as well as stimulator of interferon genes (STING) agonists have potential to enhance the innate immune response to overcome AML’s immune evasion. Immunotherapy has become a promising approach for AML treatment, especially in refractory and relapsed AML, especially in patients who are not eligible for allogeneic stem cell transplants. Future research should focus on a deeper understanding of the immune microenvironment to identify the most critical targets for optimization, as well as personalized therapeutic combination strategies. Here we present a comprehensive overview of the recent developments in immunotherapy for relapsed and refractory AML.

## 1 Introduction

**Figure 1:**
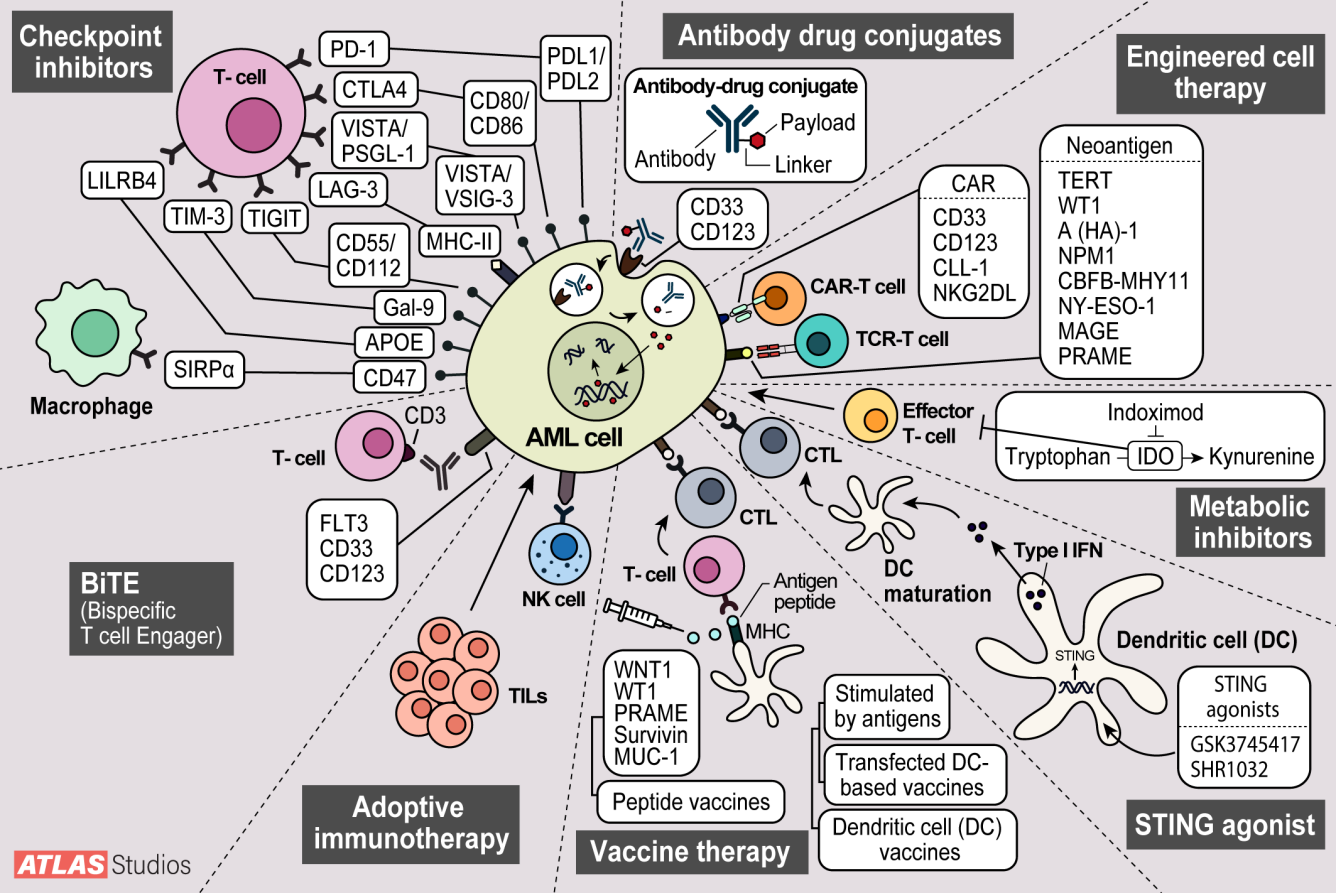
Modes of action of current AML therapy being investigated. 1. Checkpoint inhibitors stop cell cycle progression. 2. Antibody drug conjugates deliver toxic payloads to cancer cells. 3. Engineered T-cell therapy modifies t-cells to target cancer cells.4. Metabolic inhibitors inhibit metabolite degradation leading to build up in cancer cells and growth inhibition. 5. STING agonists elicit increased immune responses with increases in interferon. 6. Vaccines sensitize host immune cells to cancer antigens leading to increased immune surveillance. 7. Adoptive cell therapy enhances natural killer and tumor infiltrating lymphocytes anti-tumor effect. 8. Bispecific T-cell Engagers (BiTEs) offer another way to engage host t-cells with cancer cells.

Acute myeloid leukemia (AML) is an aggressive hematological malignancy arising from an immature myeloid progenitor primarily impacting older adults with inferior outcomes (1). The mainstay of first-line treatment for AML has been chemotherapy (2). High-dose chemotherapy, commonly known as 7 + 3, has been the standard of care for AML for years, but it is associated with high mortality and morbidity. The current regimen of 7 + 3 induction chemotherapy is then followed by consolidation chemotherapy and/or allogeneic hematopoietic stem cell transplantation (HSCT). However, 30-40% of these patients will relapse. In patients who relapse or never respond to therapy, long-term survival is only seen in approximately 30% of patients (3). Venetoclax (Ven) has an essential role in AML as it is now approved in the front-line setting in combination with azacitidine (Aza), decitabine (Dec), or low-dose cytarabine (LDAC) for patients aged 75 years and older, or those with significant comorbidities. Elderly patients have a high induction-related mortality rate of 15-30%, mainly due to infectious complications (4–7). The present approaches highlight the need for novel therapeutic approaches, including immunotherapy. Here, we review recent developments in immunotherapy for AML. Topics reviewed include checkpoint inhibitor therapy, antibody-drug conjugates, chimeric antigen receptor-engineered T cells (CAR-T), T-cell receptor-engineered T cells (TCR-T), and dendritic cell (DC) vaccines (**Figure 1**).

## 2 Checkpoint inhibitor therapy

Immune checkpoint inhibitors are regulatory molecules used to induce self-tolerance and prevent autoimmunity. These molecules are highly expressed in T cells. Studies have shown that AML cells express the ligands to these immune checkpoints to evade immune surveillance (8).

A knowledge of the microenvironment in AML is essential for understanding mechanisms of evading immune surveillance and therapeutic potential of targeting specific microenvironment components (9). Guo and colleagues analyzed major immune cell subsets in the AML bone marrow microenvironment and were able to predict prognosis in AML patients (9). The study analyzed subsets of mast cells, dendritic cells, monocytes and macrophages, and T cells. Exhausted T cells and immunosuppressive T cells like regulatory T cells (Treg) and other T cell subsets can be used as targets of anti-CTLA4, anti-PD1, and anti-CD25 therapies (9).

One of the most well-studied immune checkpoint axis is the programmed-cell-death-ligand 1 (PD-L1), which binds to PD-1 receptors expressed on T cells and thereby produces a negative costimulatory signal to attenuate T-cell activation (10). Several other immune checkpoint ligands have been recently explored as alternative targets for AML, and are reviewed below (**Table 1**) (11).

**Table 1:** Trials and studies using checkpoint inhibitors.

Trial (Identifier)	Study Name	Status	Study design	Phase	Study drug	Drug Class	Disease setting/Study Population	Results	Safety
NCT02397720	Nivolumab and Azacitidine With or Without Ipilimumab in Treating Patients With Refractory/Relapsed or Newly Diagnosed Acute Myeloid Leukemia	completed	open-label, non-randomized	II	Nivolumab and Azacitidine +/- Ipilimumab	PD-1	R/R or Newly Diagnosed AML	ORR 33%, CR/CRi 22%, median OS 6.3 months	22% grade>2 toxicities
NCT02768792	High Dose Cytarabine Followed by Pembrolizumab in Relapsed/Refractory AML	Active, not recruting	open-label	II	High Dose Cytarabine Followed by Pembrolizumab	PD-1	R/R AML	ORR 46%, CRc38%, and OS 11.1 months	Grade ≥3 immune-related adverse events were rate (14%), most common toxicities were febrile neutropenia (62%), and ALT elevation (41%).
AARON trial (NCT04913922)	Relatlimab With Nivolumab and 5-Azacytidine for the Treatment of AML	recruiting	open-label	II	nivolumab (480 mg IV day 1), azacytidine (75 mg/m2 BSA for 7 days), and relatlimab (80-160 mg IV day 1)	LAG3	R/R AML and newly diagnosed AML in elderly >/=65	n/a	n/a
(NCT03066648)	Study of PDR001 and/​or MBG453 in Combination With Decitabine in Patients With AML or High Risk MDS	completed	multi-arm, non-randomized, open-label	Ib	Decitabine, PDR001 (spartalizumab), MBG453 (sabatolimab)	TIM3	R/R AML following ≥1 prior therapies; de novo AML not candidates for std tx, but HMA-naive	Median OS after HSCT was not reached. At 2-years, RFS estimated at 64%, while OS was 69%	Acute GVHD (57%), maximum grade 3-4 aGVHD (14%), one pt died on hospice
NCT04623216	Sabatolimab as a Treatment for Patients With Acute Myeloid Leukemia and Presence of Measurable Residual Disease After Allogeneic Stem Cell Transplantation.	recruiting	open-label, multicenter	Ib/II	Sabatolimab (400 mg, 800 mg), azacitidine	TIM3	MRD+ post-aHSCT AML	n/a	n/a
NCT04372433	IO-202 as Monotherapy and IO-202 Plus Azacitidine ± Venetoclax in Patients in AML and CMML	recruiting	open-label	I	IO-202	LILRB4	R/R AML	1 patient acheived PR, 1 achieved CR	Well tolerated, no DLT.
NCT02890329	Ipilimumab and Decitabine in Treating Patients With Relapsed or Refractory Myelodysplastic Syndrome or Acute Myeloid Leukemia	active, not recruiting	non-randomized, open-label	I	Ipililumab and decitabine	PD-1	R/R AML or R/R MDS	Post-HSCT: ORR (20%), mean DOR 4.46 months. Transplant-naive: ORR (52%), mean DOR 6.14 months	Post-HSCT: neutropenia (32%), thrombocytopenia (28%), febrile neutropenia (36%), overall irAE (44%). Transplant-naive: neutropenia (48%), thrombocytopenia (48%), febrile neutropenia (61%), overall irAE (48%)
NCT03248479	The first-in-class anti-CD47 antibody Hu5F9-G4 is active and well tolerated alone or with azacitidine in AML and MDS patients	Terminated	non-randomized, open-label	Ib	Hu5F9-G4	Anti-CD47	R/R AML or MDS; previously untreated AML/MDS	CR (32.2%), CR in patients with TP53 (31.9%), median OS TP53-mutant 9.8 months, Wild-type 18.9 months	TRAE's constipation (49.4%), nausea (49.4%), diarrhea (48.3%), anemia (34.5%)
NCT04778397	Study of Magrolimab in Combination With Azacitidine Versus Physician's Choice of Venetoclax in Combination With Azacitidine or Intensive Chemotherapy in Patients With TP53 Mutant Acute Myeloid Leukemia That Have Not Been Treated (ENHANCE-2)	Terminated	randomized, open-label	III	Magrolimab	Anti-CD47	AML with TP53 mutation that have not previously been treated	Study was discontinued by researchers due to data suggestring magrolimab was unlikely to provide a survival benefit over standard care.	n/a
NCT05079230	Study of Magrolimab Versus Placebo in Combination With Venetoclax and Azacitidine in Participants With Acute Myeloid Leukemia (ENHANCE-3)	Terminated	randomized, double-blind	III	Magrolimab+ aza/ven	Anti-CD47	Untreated acute myeloid leukemia unfit for intensive therapy	Magrolimab arm of study was found to have a 17% increased risk of death compared to control arm.	There was an increased rate of AE in the Magrolimab arm including and increase in AEs leading to death (18.5% vs 10.9%). AEs included neutropenia, MI, cardiogenic shock, infection, and respiratory failure among others.
NCT04435691	Magrolimab, Azacitidine, and Venetoclax for the Treatment of Acute Myeloid Leukemia	Active, not recruiting	open-label	IB/II	Magrolimab	Anti-CD47	R/R AML ECOG ≤2	2/17 (12%) had response. mOS 3.1months, DOR 3.6	Grade ≥ grade 3 anemia in 24%, TRAE's neutropenia (50%), pneumonia (38%), hyperbilirubinemia (11%)
NCT05275439	Phase 1 Study of Shattuck Labs (SL)-172154 in Subjects with MDS or AML	recruiting	open-label	Ia/1b	SL-172154	SIRPα-Fc-CD40L	Higher-Risk MDS or AML	Relative BM blasts reduction in 2/5 patients with R/R AML at 1mg SL-AZA and 5/7 patients at 3mg SL-AZA. One patient underwent an alloHCT	SL-172154 treatment-emergent AEs (TEAs) were observed in 13 (68%) of SL-monotherapy and 8 (44%) of patients with SL-AZA combination. Most (51) TEAs observed were grade 1 or 2, except three grade 3 events. Other SL-172154-related events were most notable for AST increase (21%) and ALT increase (16%), but all events were transient.
NCT04202003	A Study to Evaluate the Safety and Tolerability, Pharmacokinetics, Pharmacodynamics and Preliminary Efficacy of TJ011133 as Monotherapy and in Combination With Azacitidine (AZA) in Patients With Acute Myeloid Leukemia (AML) or Myelodysplastic Syndrome (MDS)	Active, not recruiting	open-label	I/II	TJ011133	Anti-CD47	AML/MDS	CD47 receptor occupancy (RO) was evaluated as a PD marker. The mean RO on peripheral T cells at Cmax was 74.0%, 82.0% and 84.9% for 1, 3 and 10 mg/kg, respectively. The mean RO on CD33+ tumor cells at Cmax was 45.9%, 75.8% and 82.2% for 1, 3 and 10 mg/kg, respectively. Of note, one patient with primary refractory AML achieved morphologic leukemia-free state (MLFS) after 2 cycles of lemzoparlimab treatment at 1 mg/kg.	Lemzoparlimab was well tolerated in 5 r/r AML pts in the doses tested and importantly, consistent with preclinical data, there were no serious hematological AEs so far. No DLTs or MTD was observed up to 10 mg/kg weekly dosing so far.

### 2.1 PD-1/PD-L1

As described, PD-1, programmed death-ligand 1, is the most developed immune checkpoint inhibitor investigated in AML. Commonly used drugs in this drug class include nivolumab, pembrolizumab, and atezolizumab. Increased expression of PD-1 has been documented in subsets of populations such as relapsed/refractory (R/R) AML and demonstrates poorer outcomes. Therefore, patients with increased expression of PD-1 benefit from this immune checkpoint inhibitor therapy. Hypomethylating agents (HMA) are also used in the therapy of AML. These drugs work by inhibiting methyltransferase, altering gene expression (12). Recent studies have identified a correlation between HMA resistance and dysregulated PD-1/PD-L1 signaling. Higher levels of expression of PD-1/PD-L1 have been seen in patients with AML with no response to HMAs, which may represent a potential mechanism of resistance to this class of agents. Therefore, the efficacy of monoclonal antibodies targeting this pathway in HMA-failure myelodysplastic syndrome (MDS) or R/R AML is assessed. A nonrandomized, open-label phase II study by Daver et al. targeted PD-1 in combination with HMA in R/R AML [both HMA naïve (n=25) and HMA failure (n=45)] with nivolumab and azacitidine. They observed an overall response rate (ORR) of 33% (58% in HMA-naive and 22% in HMA-failure patients). Complete remission with incomplete count recovery (CR/CRi) was 22%, and median overall survival (OS) was 6.3 months with 22% grade >2 immune toxicities (13).

Pembrolizumab has also been evaluated in both newly diagnosed and R/R AML patients in combination with azacitidine by Gojo and colleagues (14). In the R/R cohort, the ORR was noted to be 18%, with 14% of patients experiencing CR/CRi and PR in 4% of patients with a median OS of 10.8 months for all patients. However, in newly diagnosed patients, the ORR was 59% (CR/CRi 47%; PR 12%) with a median OS of 13.1 months. Additionally, pembrolizumab has been evaluated with decitabine in R/R AML. This study was limited by a small number of patients (n=10). Stable disease was achieved by 4 patients, with 1 patient experiencing minimal residual disease and a negative CR. The median follow up was 13 months, with median OS of 7 months.

The role of PD-L1 blockade in combination with HMA in patients with AML has also been explored (15). Zeidan and colleagues randomized patients with newly diagnosed AML to receive azacitidine alone (n=64) vs. azacitidine in combination with durvalumab (n=65). The ORR was similar in both arms, with ORR of 31.3% in patients receiving azacitidine alone and ORR of 35.4% in patients receiving combination therapy. Unfortunately, no difference was observed in OS or duration of response between groups. The OS and duration of response for patients treated with single agent HMA were 13.0 months and 24.6 weeks, with the doublet cohort experiencing OS of 14.4 months and duration of response 51.7 weeks.

Lastly, intensive chemotherapy has also been evaluated in combination with anti-PD-1 therapy. A phase II single arm clinical trial conducted by Zeidner et al. examined PD-1 inhibition using pembrolizumab in combination with intensive chemotherapy (high dose cytarabine) in R/R AML. These authors demonstrated an ORR of 46% in the 37 patients treated with 38% of patients experiencing a composite complete remission rate, and a median OS of 11.1 months (15). Although much fervor has surrounded agents targeting PD-1 and PD-L1 in other malignancies due to impressive and durable responses, enthusiasm has waned for utilizing these agents in AML as a result of minimal improvement in responses and survival.

### 2.2 TIGIT

TIGIT, or T cell immunoreceptor with immunoglobulin and ITM domain; is a promising immune checkpoint (11). TIGIT is an immune checkpoint receptor part of the immunoglobulin (Ig) superfamily and is regulated by a plethora of immune cells such as NK cells, activated T cells, and regulatory T cells. CD155 and CD112 are expressed on tumor cells and antigen-presenting cells (APC) within the tumor microenvironment. TIGIT has two binding sites for these CD antigens. Studies have shown that TIGIT plays a role in regulating T and NK cell-mediated recognition. Although TIGIT inhibition alone has shown less impressive antitumor effects, dual blockade of TIGIT with PD-1 has demonstrated almost complete tumor rejection in murine tumor models, which is supportive of further investigation in patients with cancer.

Prior studies have found that the microenvironment of the leukemia bone marrow contains immunosuppressive factors promoting the evasion of leukemic stem cells from immunological surveillance. In a 2021 Xu L et al. study, findings showed higher co-expression of PD-1 and TIGIT in both CD4+ and CD8+ T cells in bone marrow samples of *de novo* AML patients compared to peripheral blood samples, supporting that there are increased immunosuppressive factors in bone marrow compared to peripheral blood (**Table 2**) (16).

**Table 2:** Trials and studies using other antibody therapies such as antibody drug conjugates and bispecific t-cell engagers.

Trial (Identifier)	Study Name	Status	Study design	Phase	Study drug	Drug Class	Disease setting/Study Population	Results	Safety
NCT00401739	Safety, Tolerability, and Pharmacokinetics of CSL360 in the Treatment of Acute Myeloid Leukemia	Completed	open-label	I	CSL360	Anti-123	R/R AML, High-Risk Acute AML	28/30 patients (93.3%) had persistent disease after four hours of CSL360 according to NCCN response criteria	TRAE's headache (32.5%), diarrhea (27.5%), nausea (27.5%), and infection (17.3%).
NCT04429191	JSP191 Antibody Conditioning Regimen in MDS/​AML Subjects Undergoing Allogenic Hematopoietic Stem Cell Transplantation	Completed	open-label	I	Briquilimab (JSP191)	Anti-CD117	AML	Of 13 patients in CR prior to HSCT, 9 were alive at 1 year, OS 69.2%, 8 of those, 61.5%, were MRD negative. All patients engrafted with one requiring a second transplant due to secondary graft failure.	Well tolerated, no serious AEs related to briquilimab. All patients had some degree of GCHD with one death due to gastrointestinal GVHD.
NCT03386513	Clinical Profile of IMGN632, a Novel CD123-Targeting Antibody-Drug Conjugate (ADC), in Patients with Relapsed/Refractory (R/R) Acute Myeloid Leukemia (AML) or Blastic Plasmacytoid Dendritic Cell Neoplasm (BPDCN)	Active	open-label	I	IMGN632	Anti-CD123	AML	Preliminary results from 66 patients with R/R AML, had 55% showing a reduction in bone marrow blasts with 20% achieving CR (3), CRi (8), or MLFS (2)	Among the 74 patients evaluated between the two groups, the most common AEs were diarrhea (30%), febrile neutropenia (27%), nausea 20%, chills (23%) and lung infection (22%). Three dose limiting toxicities occurred, one prolonged neutropenia and two reversible instances of VOD.
NCT04086264	A Phase 1b/2 Study of IMGN632, a CD123-Targeting Antibody-Drug Conjugate (ADC), As Monotherapy or in Combination with Venetoclax and/or Azacitidine for Patients with CD123-Positive Acute Myeloid Leukemia	Active	open-label	Ib/II	IMGN632	Anti-CD123	CD123+ AML	n/a	n/a
NCT03647800	Study of APVO436 in Patients With AML or MDS	recruiting	open-label	I/Ib	APVO436-5001	Anti-CD123 x Anti-CD3 ADAPTIR	AML/MDS	Of the 34 relapsed AML patients evaluable for surrogate response measurements, 8 had clinically meaningful stabilization of their leukemia or a CR that lasted >3 months.	With 46 patients with either R/R AML (39) or MDS (7) studied, APVO436 exhibited a favorable safety profile. The most common APVO436-related AEs were infusion-related reactions (IRR) occurring in 13 (28.3%) patients and cytokine release syndrome (CRS) occurring in 10 (21.7%).
NCT02520427	A Phase 1 First-in-Human Study of AMG 330, an Anti-CD33 Bispecific T-Cell Engager (BiTE®) Antibody Construct, in Relapsed/Refractory Acute Myeloid Leukemia (R/R AML)	Terminated	open-label	I	AMG 330	Anti-CD33	R/R AML	Out of 35 patients, 5 showed response with 2 in CR, 2 in CRi and one in a morphological leukemia free state.	23 adverse events occurred 15 of which were treatment related. Most commonly cytokine release syndrome (11), febrile neutropenia (6), pneumonia (4), leukopenia (3) and thrombocytopenia (2).

Brauneck and colleagues identified TIGIT as a target for macrophage-mediated cytotoxicity in AML. TIGIT was highly expressed on bone marrow-infiltrating immunosuppressive M2 macrophages within newly diagnosed and relapsed AML. Additionally, they found that *in vitro* blockade of TIGIT shifted the polarization of leukemic-associated macrophages from the M2 phenotype to the M1 phenotype. They also reported anti-CD47-mediated phagocytosis of AML cells augmented by TIGIT blockade (17).

Gournay and colleagues also demonstrated that high levels of TIGIT on CD4+ T cells three months after allogeneic HSCT was significantly associated with an increased risk of subsequent relapse in AML patients. Additionally, higher expression levels of TIGIT on CD8+ T cells were directly correlated with poorer clinical outcomes (18). These findings support that this immune checkpoint inhibitor is important in post-transplant relapse (19). Overall, TIGIT represents a potential target in AML. However, data supporting the pre-clinical or clinical activity of an agent targeting TIGIT remains to be seen.

### 2.3 LAG3

Lymphocyte activation gene 3 (LAG3) is a co-inhibitory receptor belonging to the Ig superfamily, also known as CD223, which may be another promising immune checkpoint therapy for AML immunosuppression (20). It suppresses T cell activation and cytokine secretion and demonstrates synergism with PD-1 to inhibit an immune response. LAG3 is a promising target as it has shown efficacy against PD1-resistant AML in conjunction with PD1 therapy. Anti-LAG3 antibodies can upregulate effector T-cell activity and inhibit T-regulatory cell suppressive function.

In the 2019 study by Abdelhakim, H. et al., these authors found that AML cells promoted immunosuppression with higher expression of LAG3 on CD8+ T cells the *in vitro* model. Peripheral blood mononuclear cells were harvested from healthy donors and AML patients (**Table 2**). Anti-LAG3 antibody attenuated this immunosuppression by increasing the number of activated T cells and cytotoxic cytokines, decreasing T-reg cells, and enhancing MHC-I mediated killing of AML cells, thus supporting the inhibition of LAG3 as a means to mitigating AML immunosuppressive effect (21).

Chen, C. et al. investigated the expression pattern of LAG3 and other immune checkpoints in AML using data from *de novo* AML patients within the TCGA database and AML bone marrow samples (**Table 2**). They found that AML patients had poorer overall survival with high CTLA-4 and LAG3 expression (3-year OS 9% vs. 36% and 13% vs. 40%, respectively) (22).

Relatlimab is a monoclonal IgG4 antibody that blocks LAG-3. The Food and Drug Administration (FDA) recently approved it as a treatment for untreated advanced malignant melanoma. The AARON trial (NCT04913922) is a phase 2, open-label clinical trial within its recruiting phase to investigate the safety and efficacy of combination therapy with nivolumab, azacitidine, and relatlimab in R/R AML (**Table 1**) (23). The results from this clinical trial will be pivotal in understanding whether targeting LAG-3 can enhance the activity of anti-PD-1 therapy in AML when combined with HMA.

### 2.4 TIM-3

T cell immunoglobulin and mucin domain 3, TIM3, is a cell surface molecule expressed on leukemia cells (24). In CD4+ and CD8+ T cells and leukemic cells, TIM3 overexpression may lead to poor response and relapse in patients; therefore, TIM3 inhibitors are currently being investigated as potential AML treatments. Regarding acute myeloid leukemia, studies have shown that leukemia stem cells (LSC) have higher expression of TIM3 but not in healthy hematopoietic stem cells (HSC). Therefore, targeting TIM3 with antibodies or microRNA can destroy LSCs without affecting healthy HSCs. In leukemic cells of AML, TIM3 participates in an autocrine stimulatory loop, which activates the β-catenin and NF-κB pathways integral for cell survival and disease progression. Moreover, TIM3 inhibitors induce cell apoptosis in AML (24).

Additionally, Al-Amrani, S. et al. (2022) have shown that there is a higher expression of TIM3 in peripheral blood of AML cell lysates compared to healthy controls with median concentration 9.26 pg/ml and 5.1 pg/ml respectively (p=0.01) (**Table 2**) (25). TIM3 concentrations were also significantly elevated at diagnosis compared to remission, 9.25 pg/ml and 5.57 pg/ml, respectively (p=0.03). In bone marrow samples, TIM3 expression was higher in AML samples than in healthy controls, 10.89 pg/ml and 5.1 pg/ml, respectively, at diagnosis (p=0.04). The expression levels of TIM3 from AML bone marrow lysates at diagnosis and in remission were not significantly different (10.8 pg/ml and 5.1 pg/ml, p=0.01).

A multi-arm open-label phase 1b study (NCT03066648) investigates combination therapy with sabatolimab (MBG453), which is an anti-TIM-3 IgG4 and a hypomethylating agent (decitabine or azacitidine) (26). Preliminary findings with just sabatolimab have shown promising survival rates in R/R AML and *de novo* AML patients who are not candidates for standard treatment (HMA-naive) (**Table 1**). An analysis was performed on 28 of these patients who underwent an allogeneic HSCT. Before HSCT, 36% of patients had a complete response, and 32% had a complete molecular or complete response without hematologic recovery. 8% had a partial response or hematologic improvement, and 25% had stable disease. After allogeneic HSCT, median OS was not reached. However, the 2-year OS rate was 69%, and the 2-year recurrence-free survival rate was 64%. Regarding adverse effects, 57% experienced acute graft-vs-host disease (GVHD), 14% of which experienced grade 3/4 GVHD, where one patient died in hospice after grade 4 GVHD.

A phase 1b/2, open-label study (NCT04623216) currently recruiting aims to investigate sabotolimab monotherapy in combination with azacitidine in AML patients who received one allogeneic HSCT achieving complete remission but were minimal residual disease positive (MRD+ post-allogeneic HSCT) (**Table 1**) (27). Results from 21 patients showed 7 patients were in complete remission (CR) and on treatment after one year of therapy. The remaining 14 had been discontinued on treatment due to relapse, AE, or treatment change. There were toxicities, with 76% of patients experiencing any toxicity and 38% experiencing grade 3 or higher toxicities. Most commonly, neutropenia and thrombocytopenia. There were 4 patients who experienced dose limiting toxicities. A follow-up dose expansion study is planned (28). Unfortunately, although there was much enthusiasm for this compound, in January 2024, Novartis discontinued the program evaluating sabatolimab in high-risk myelodysplastic syndromes as the STIMULUS-MDS trial did not meet its primary endpoints of improved complete response rates or progression-free survival (29).

### 2.5 VISTA

VISTA (V-domain Ig suppressor of T cell activation) is a novel immune checkpoint similar to PD-1 or CTLA-4. VISTA is highly expressed in primary AML cells and is associated with a poor prognosis (30). Based on the TCGA database, VISTA was the most correlative immune checkpoint in the overall prognosis of AML patients as it had the same higher expression as in CD34+ AML cells and CD34- myeloid cells of bone marrow. VISTA helps impede T-cell activation. Hyperactive STAT3 in AML has been associated with a high expression of VISTA. One STAT3 inhibitor, W1046, suppresses AML proliferation and survival. The inhibition of STAT3 causes a downregulation of VISTA, which allows for activation of T cells. However, the molecular mechanism of how this occurs is unknown. T-cell-mediated cytotoxicity to AML cells is also boosted through anti-VISTA mAb, and anti-VISTA mAb was also shown to prolong the survival of AML in mice*. In vitro* and *in vivo* studies have also shown that the combination of W1046 and VISTA mAB caused a drastic elevation in T cell cytotoxicity *in vitro* and also caused upregulation of the secretion of interferon (IFN)-y, interleukin (IL)-2, and CD4+ and CD8+ T cells. Although VISTA has been more thoroughly studied in other malignancies, the role of targeting this checkpoint as an anti-leukemic therapeutic strategy remains to be seen.

### 2.6 Anti-CD47

CD47 was initially discovered as an antigen in human ovarian cancer and later found to be overexpressed in hematological tumors like AML (31). CD47 supports cells in evading phagocytosis by macrophages in leukemic cancer cells, and much enthusiasm has been put behind this pathway as evidenced by multiple compounds being developed to target this checkpoint in AML (**Table 1**).

#### 2.6.1 CD47: SL-172154 (SIRPα-Fc-CD40L)

CD47 blocking antibodies and SIRPα-FC fusion proteins have been studied in combination with azacitidine in untreated AML patients. SL172154 is a CD47 inhibitor that has enhanced pro-phagocytic signals on leukemic stem cells/blats coupled with CD40L to enhance antigen presentation and further enhance tumor cell killing (32). A phase I dose escalation trial is evaluating SL-172154 as monotherapy vs in combination with azacitidine for patients with AML or HR-MDS. The study had 37 patients enrolled, with 27 patients having R/R AML (16 *de novo*; 11 secondary). SL-172154 treatment-emergent AEs (TEAs) were observed in 13 (68%) of SL-monotherapy and 8 (44%) of patients with SL-AZA combination. Most (51) TEAs observed were grade 1 or 2, except three grade 3 events. Other SL-172154-related events were most notable for AST increase (21%) and ALT increase (16%), however all events were transient.

Overall, one patient with R/R AML post 7 + 3, FLAG, and VEN/AZA achieved a Morphologic Leukemia-Free State (blast reduction 19% down to 5%) after 1 cycle 6mg/kg SL-172154 and proceeded to allogeneic HCT after cycle 2 (32). There were 12 patients with R/R AML who received SL-AZA with no objective responses observed, but the reduction in bone marrow blasts from baseline (50-75%) was seen in 2/5 patients at 1mg SL-AZA. Relative bone marrow blast reduction (35-90%) was seen in 5/7 patients at 3mg SL-AZA. One patient proceeded to allogeneic HCT.

The study demonstrated that SL-172154 is well tolerated in up to 3mg/kg monotherapy and in combination with azacitidine. There was an increase in accumulation of mature myeloid cells in BM and an increase in serum cytokines with the dose-dependent increases, which suggests a potential role for CD40 stimulation in AML (32).

#### 2.6.2 TJ011133 (TJC4, Lemzoparlimab)

Lemzoparlimab (TJ011133 or TJC4) is a differentiated anti-CD47 IgG4 antibody that targets a CD47 epitope, which allows a unique red blood cell sparing property while retaining strong anti-tumor activity (33). It is currently being evaluated in a phase I trial (NCT04202003). The unique epitope differentiates it from other CD47 axis targeting therapies. Lemzoparlimab blocks CD47-SIRPα interaction, which helps promote phagocytosis of cancer cells by macrophages and neutrophils (34).

Dosages of 1mg/kg, 3mg/kg, and 10mg/kg were used with average receptor occupancy on peripheral T cells of 74%, 82%, and 84% respectively. One patient attained morphologic leukemia-free status after two cycles of 1mg/kg, but 4/5 patients had AE, with 1/5 being a Grade 3 (35).

#### 2.6.3 Magrolimab (Hu5F9-G4)

Hu5F9-G4 was analyzed in a clinical trial (NCT0324879) where 53% (8/15) untreated AML/MDS patients had either CR or Cri (36). Another phase Ib trial (NCT04778397), showed 56% (10/34) AML patients achieved CR/CRi to 5F9+AZA, and CR/CRi rate was greater in patients with TP53 mutations (36). Another study showed in relapsed refractory AML with prior venetoclax-naive AML, the CR/CRi rate was 63% (5/8) (37). In relapsed refractory AML with prior Venetoclax failure, the CR/CRi rate was 27% (3/13) with a median OS of 3.1 (37).

While CD47/SIRPα immune checkpoint-based immunotherapy studies have shown promising preliminary results in R/R AML, the development of CD47 monoclonal antibodies still faces safety concerns and a lack of published data on therapeutic effectiveness. One area of concern is the significant dosage requirement to achieve adequate therapeutic CD47 blockage, as a preclinical study showed that 40-60% of CD47 receptor occupancy is required for the induction of phagocytosis (38). With this in mind, magrolimab, Gilead’s CD47/SIRPa interaction blocking monoclonal antibody being investigated in their phase 3 ENHANCE-3 study, was recently terminated in February of 2024. An independent data analysis demonstrated evidence of futility within the magrolimab, azacitidine, and venetoclax arm which resulted in an increased risk of death due to infection and respiratory compromise. The FDA ultimately paused enrollment applicable to all studies of magrolimab. Gilead will not be pursuing magrolimab further in hematological cancers. With this large multi-billion-dollar study ultimately failing to demonstrate any benefit, the future of CD47 drug therapy is uncertain, even if ways are found to minimize toxicity.

### 2.7 LILRB4

LILRB4 is a member of the leukocyte immunoglobulin-like receptor family of proteins commonly seen on myeloid lineage cells. They play an important part in cell activation and differentiation. LILRB4 is seen most commonly in monocytic AML. Working through APOE, SHP-2, uPAR, and ARG1 signaling pathways, LILRB4 activation leads to downregulation of T cell activation, aiding in cancer infiltration and immune system evasion (39). Similar to other targets discussed, LILRB4 overexpression in cancer cells compared to normal cells makes it an enticing target for immune checkpoint inhibition and other therapies. NCT04372433 is an active Phase 1 trial evaluating IO-202, a humanized monoclonal antibody blocking LILRB4 from binding to APOE and fibronectin (**Table 1**). Part 1 of the study reported on 36 patients with R/R AML with monocytic differentiation treated with azacitidine ± Venetoclax or as monotherapy. Overall, the drug was well tolerated, with no dose-limiting toxicity observed and no study-related deaths. Unfortunately, in the monotherapy group, only one patient achieved a partial remission. In the combination group, only one patient (noted to have high LILRB4 expression) achieved CR, which was durable for 7 months as of the report. Several other patients did show blast reduction but did not achieve remission. While this trial showed limited success in general monocytic AML, patients were not screened for high LILRB4 expression. Part two of the trial will expand to look at patients with high LILRB4 expression which will hopefully yield more promising results (40). There are two other up-and-coming trials using STAR-T or CAR-T cells targeting LILRB4 that have been indexed; however, they have not started recruiting or been reported on.

## 3 Monoclonal antibody therapy

### 3.1 Anti-CD123

CD123 is a cytokine receptor widely overexpressed in multiple hematologic cancer cells and associated with high-risk disease characteristics in adult and pediatric AML. In particular, AML cells with FLT-3 or NPM1 mutations are associated with higher levels of CD123 compared to cells with wild-type FLT3 or NPM1 (41). CD123 has become an important biomarker that can be potentially targeted in relapsed or refractory AML. CSL360 is a recombinant chimeric immunoglobulin G1 (IgG1), an anti-CD123 monoclonal antibody proven less beneficial in patients with relapsed/refractory or high-risk AML (42, 43). The phase I clinical trial (NCT00401739) assessed the efficacy and safety of CSL360 in relapsed/refractory AML cases (**Table 3**). The study analyzed 40 patients, with 25 of the patients having relapsed AML and 8 of the patients having refractory disease. The study found that CSL360 was well tolerated, as the most common AEs of any grade were headache (32.5%), diarrhea (27.5%), nausea (27.5%), and infection (17.3%). AEs also did not increase the frequency of the dose level escalation. The study found no anti-leukemic effects as most patients had no improvement in blast percentage in BM aspirates during the study. The study found that 28/30 patients (93.3%) had persistent disease after four hours of CSL360, according to NCCN response criteria (42).

**Table 3:** Trials and studies using engineered cells and adoptive therapy.

Trial (Identifier)	Study Name	Status	Study design	Phase	Study drug	Drug Class	Disease setting/Study Population	Results	Safety
NCT02159495	Genetically Modified T-cell Immunotherapy in Treating Patients With Relapsed/​Refractory Acute Myeloid Leukemia and Persistent/​Recurrent Blastic Plasmacytoid Dendritic Cell Neoplasm	Active, not recruiting	open-label	I	CD123	CAR-T-Cell Therapy CD 123	Relapsed/​Refractory or Recurrent Blastic Plasmacytoid	n/a	n/a
NCT03190278	Ameli-01: A Phase I Trial of UCART123v1.2, an Anti-CD123 Allogeneic CAR-T Cell Product, in Adult Patients with Relapsed or Refractory (R/R) CD123+ Acute Myeloid Leukemia (AML)	Recruiting	open-label	I	UCART (anti-CD123)		Relapsed/Refractory AML	Preliminary	Cytokine release syndrome (CRS) occurred in 15/16 patients (FC arm [n=7]; FCA arm [n=8]), of which 3 patients experienced ≥ G3 CRS which were classified as DLTs as noted above.
NCT03126864	Study of Adoptive Cellular Therapy Using Autologous T Cells Transduced With Lentivirus to Express a CD33 Specific Chimeric Antigen Receptor in Patients With Relapsed or Refractory CD33-Positive Acute Myeloid Leukemia	Completed	open-label	I	CD 33	CD33-CAR-T	Relapsed/Refractory or CD-33 Positive AML	No anti-leukemia response seen	No DLTs. 2 patinets experience cytokine release syndrome (CRS) and 1 developed immune effector cell-associated neurotoxicity syndrome
(NCT04351022)	Pilot Study of the Efficacy and Safety of CD38 Targeted Chimeric Antigen Receptor Enginerred T-Cells in the Treatment of CD38 Positive Relapsed or Refractory Acute Myeloid Leukemia (AML)	Completed	open label	I/II	CD38	CD38-CAR-T	R/R AML	1/6 patients achieved blast reduction from 15.5-5%, remaining 5 had no response. 4/6 patients achieved CR at 4 weeks.	1 patient expereinced Grade III hepatotoxicity. Neutropenia was persistent during CAR-T-38 therapy
NCT05467202	Evaluate the Safety and Efficacy of CLL1 CAR-T in Patients with R/R AML	Not yet recruiting	open label	I	CLL1	CLL1 CAR-T	R/R AML	70% of patients observed complete response	All patients developed cytokine relsease syndrome (4 low grade, 6 high-grade); all experienced severe pancytopenia, and two patients dieddue to infection
NCT05467202	Evaluate the Safety and Efficacy of CLL1 CAR-T in Patients with R/R AML	Not yet recruiting	open label	I	CLL1	CLL1 CAR-T	R/R AML	70% of patients observed complete response	All patients developed cytokine relsease syndrome (4 low grade, 6 high-grade); all experienced severe pancytopenia, and two patients dieddue to infection
NCT02203825	Phase I Trial of Autologous CAR T Cells Targeting NKG2D Ligands in Patients with AML/MDS and Multiple Myeloma	Completed	open label	I	NKG2DL	NKG2DL CAR-T	acute myeloid leukemia/myelodysplastic syndrome or relapsed/refractory multiple myeloma	With a single injection of low cell doses used in this trial, no objective tumor responses were observed. However, hematologic parameters transiently improved in one subject with AML at the highest dose, and cases of disease stability without further therapy or on subsequent treatments were noted. At 24 hours, the cytokine RANTES increased a median of 1.9-fold among all subjects and 5.8-fold among six AML patients. Consistent with preclinical studies, NKG2D-CAR T cell–expansion and persistence were limited. Manufactured NKG2D-CAR T cells exhibited functional activity against autologous tumor cells in vitro, but modifications to enhance CAR T-cell expansion and target density may be needed to boost clinical activity.	No dose-limiting toxicities, cytokine release syndrome, or CAR T cell–related neurotoxicity was observed. No significant autoimmune reactions were noted, and none of the ≥ grade 3 adverse events were attributable to NKG2D-CAR T cells.
(NCT03018405)	A Dose Escalation Phase I Study to Assess the Safety and Clinical Activity of Multiple Cancer Indications	Completed	open label	I	CYAD-1	CAR-T		25% ORR; 2 patients prcoeeded to HSCT	44% had grade 3-4 TRAE. 31% had grade 3-4 CRS. One DLT of CRS was reported at dose level 3. No treatment related deaths occurred. Maximum tolerated dose was not reached.
NCT02203825	Phase I Trial of Autologous CAR T Cells Targeting NKG2D Ligands in Patients with AML/MDS and Multiple Myeloma	Completed	open label	I	NKG2DL	NKG2DL CAR-T	acute myeloid leukemia/myelodysplastic syndrome or relapsed/refractory multiple myeloma	With a single injection of low cell doses used in this trial, no objective tumor responses were observed. However, hematologic parameters transiently improved in one subject with AML at the highest dose, and cases of disease stability without further therapy or on subsequent treatments were noted. At 24 hours, the cytokine RANTES increased a median of 1.9-fold among all subjects and 5.8-fold among six AML patients. Consistent with preclinical studies, NKG2D-CAR T cell–expansion and persistence were limited. Manufactured NKG2D-CAR T cells exhibited functional activity against autologous tumor cells in vitro, but modifications to enhance CAR T-cell expansion and target density may be needed to boost clinical activity.	No dose-limiting toxicities, cytokine release syndrome, or CAR T cell–related neurotoxicity was observed. No significant autoimmune reactions were noted, and none of the ≥ grade 3 adverse events were attributable to NKG2D-CAR T cells.
(NCT03018405)	A Dose Escalation Phase I Study to Assess the Safety and Clinical Activity of Multiple Cancer Indications	Completed	open label	I	CYAD-1	CAR-T		25% ORR; 2 patients prcoeeded to HSCT	44% had grade 3-4 TRAE. 31% had grade 3-4 CRS. One DLT of CRS was reported at dose level 3. No treatment related deaths occurred. Maximum tolerated dose was not reached.
NCT01898793	Cytokine-induced Memory-like NK Cells in Patients With Acute Myeloid Leukemia (AML) or Myelodysplastic Syndrome (MDS)	Terminated due to insufficient funds	non-randomized, open-label	I/II	n/a	cytokine-induced ML NK	R/R AML or R/R MDS	7/15 evaluatable patient achieved CR with median leukemia free survival of responders being 84 days.	All toxicites associated with treatment were grade 1-2. No dose limiting toxicities occured. No deaths were attributable to treatment and no GVHD or CRS was seen.
NCT02782546	Cytokine Induced Memory-like NK Cell Adoptive Therapy After Haploidentical Donor Hematopoietic Cell Transplantation	Recruiting	non-randomized, open-label	II	n/a	cytokine-induced ML NK	R/R AML	13/15 patients that were evaluated achieved CR at day +28 of treatment. One additonal achieved CRi at day +44. Median event-free survival among all 15 patients was 3.2 months (range, 1.2 to 15.4). 12 patients were alive by day 100, of which four remained in composite CR at that time. Median OS was 7.0 months (range, 1.2 to 27.5), with 29% 1-year OS.	One patient developed grade 1 cytokine release syndrome (CRS), and six patients experienced grade 1 or 2 injection site reactions, none of which led to treatment modifications. Ten (67%) patients developed acute GvHD (grade 1: 4, grade 2: 6). No patient developed steroid-refractory acute GvHD. Two of 10 (20%) evaluable patients developed chronic GvHD, 1 of which was mild and only involved the skin, whereas another patient had moderate skin and gastrointestinal involvement. These rates of GvHD were comparable to expected rates with haplo-HCT, with no exacerbations attributed to ML NK cells or N-803. Two patients experienced nonrelapse mortality (NRM) in the setting of grade 5 AEs. One patient suffered primary graft failure and died from related complications on day +43; another died on day +37 from complications of sepsis.

CD123 antagonistic peptides assembled with nanomicelles have also been evaluated preclinically (**Table 2**) (16). In the study, nanomicelles were loaded with a lab-designed CD123 antagonistic peptide (mPO-6) and were investigated with CD123+ AML cell lines and a refractory AML mouse. Results showed that IV administration of mPO-6) reduced the percentage of AML cells and prolonged median survival of AML mice. It was determined that mPO-6 interferes with the axis of CD123/IL-3, which helps enhance apoptosis and prolong the median survival of AML mice.

Another preclinical study investigated SIRPα-αCD123 fusion antibodies targeting CD123 with blockade of CD47 to augment anti-CD123 activity (**Table 2**) (44). As reviewed previously, leukemic cells have CD47, an innate immune checkpoint upregulated on the AML cell surface. The authors developed a SIRP α-αCD123 fusion antibody that enhances the leukemic stem cell clearance by disrupting CD47/SIRPα signaling to AML. *In vitro*, blocking assays were used to interfere with the binding of SIRPα. The SIRP α-αCD123 fusion antibody showed increased binding and targeting of CD123+/CD47+ AML cells, even in CD47+ healthy cells. *In vitro* experiments also showed SIRPα-αCD123 antibodies greatly enhanced AML cell phagocytosis mediated by allogeneic and autologous macrophages. Unfortunately, due to a lack of clinical response, targeting AML via monoclonal antibody therapy has lost enthusiasm.

## 4 Antibody-drug conjugates

Antibody-drug conjugates (ADCs) combine the specificity of an antibody with a potent drug to bind and kill target cells. Targets for ADCs currently investigated in preclinical or early Phase I/II trials for AML include CLL-1, CD123 (also known as interleukin 3 receptor alpha), CXCR4, and FLT3. More studied targets like CD33 are already observing success in select patient populations.

### 4.1 CD33

CD33 is a receptor protein primarily expressed on leukemic blasts, and low expressions of CD33 have been seen in more complex karyotypes and translocations. Gemtuzumab ozogamicin (GO), a humanized anti-CD33 monoclonal antibody conjugated with calicheamicin, was first approved by the FDA in 2000 (45). However, the randomized phase III S0106 trial that evaluated GO versus no GO during induction and post-consolidation therapy in younger AML did not confirm promising results in the previous phase II study conducted on relapsed older adults with AML (46). The drug was withdrawn from the US market due to an unfavorable safety-efficacy profile. However, it was later placed back on the market in 2017 after multiple randomized controlled trials showed an improved OS as well as event-free survival when GO therapy is used in addition to induction/consolidation therapy in newly diagnosed AML patients with a safe toxicity profile.

The improvement of OS was further characterized when evaluating the cytogenetic profile of treated patients. One meta-analysis looked at five large, randomized trials, which analyzed GO in induction/consolidation chemotherapy in all ages of AML patients. Beneficial effects of GO were seen in favorable risk AML, along with a smaller benefit in intermediate-risk AML and no benefit in adverse-risk (47). Favorable cytogenetics saw an overall survival benefit regardless of age, 30.7% vs 34.6%. The survival benefit was mainly seen in the favorable cytogenetics (55.3% vs. 76.3%) and intermediate-risk patients (34.1% vs. 39.4%). Patients with adverse karyotypes did not see any benefit in overall survival.

Hepatic veno-occlusive disease (VOD), which is a clinical syndrome characterized by jaundice, painful hepatomegaly, and/or fluid retention, was reported in initial studies with GO in AML. Late manifestations of hepatic VOD include ascites and encephalopathy. In the meta-analysis, fractionated dosing using 3 mg/m2 was associated with less toxicity and equal efficacy (47). The data obtained from the fractionated dosing schedule led to the reapproval of GO (48).

### 4.2 CLL-1

C-type lectin-like molecule-1 (CLL-1) is primarily expressed on granulocytes and monocytes and, to a lesser degree, on precursors and HSCs. However, expression is seen in a large percentage of AMLs, including leukemic stem cells (49). Daver et al. evaluated the ADC DCLL9718S in 2021 in a phase I dose-escalation trial. DCLL9718S is a humanized monoclonal IgG1 anti-CLL1 antibody linked with two pyrrolobenzodiazepine (PBD) dimer drugs via a cleavable disulfide linker. Eighteen patients were treated in the study, and almost all participants experienced at least a grade 1 adverse event, with at least half being attributable to treatment. The most common adverse events were neutropenia and pneumonia, although no dose-limiting toxicity occurred, and no deaths occurred because of therapy. At the highest dose of 160 μg/kg, 2 patients began experiencing hepatotoxicity, although this may be intrinsic to PBD and not drugs targeting CLL-1. No patient achieved a complete or partial response. The trial was stopped, and there are no plans to move forward with the drug, given the poor tolerability and limited anti-leukemic effects observed (50). Several other groups have published their preclinical work as the potential of CLL-1 remains promising despite the setbacks seen with DCLL9718S.

### 4.3 CD123

CD123, also known as interleukin 3 receptor alpha, is another target with elevated expression in AML with the potential to target leukemic stem cells that been targeted by multiple groups. Kuvtun et al. have worked to change the cytotoxic drug component of ADCs. IMGN632 comprises a novel humanized anti-CD123 antibody, G4723A, linked to an alkylating agent, indolinobenzapine (IGN) pseudodimer (51). They previously had a DNA crosslinking agent, but as with several ADCs, toxicity became a limiting factor. IMGN632 showed potent activity against AML samples *in vitro* and in xenograft mouse models made from EOL-1, MOLM-13, and patient-derived samples (51). Substantially less toxicity to normal hematopoietic cells was seen. Daver et al. began a phase 1 clinical trial looking at IMGN632 in R/R AML and blastic plasmacytoid dendritic cell neoplasm, NCT03386513 (52). Of the 66 patients with R/R AML treated on protocol, 55% demonstrated a reduction in bone marrow blasts, with 20% achieving CR (N=3), CRi (N=8), or morphologic leukemia-free state (N=2). Among the 74 patients evaluated between the two groups, the most common AEs were diarrhea (30%), febrile neutropenia (27%), nausea 20%, chills (23%), and lung infection (22%). Three dose limiting toxicities occurred, one prolonged neutropenia and two reversible instances of VOD (53). Given the activity in the R/R setting, this combination is being explored in newly diagnosed AML patients expressing CD123 (54).

Talacotuzumab, a humanized CD123 monoclonal antibody, has shown potent *in vitro* ADCC against AML blasts, reducing leukemic cell growth in murine xenograft models (55). A multicenter phase 2/3 clinical study investigates talacotuzumab to determine the recommended phase 2 dose in AML patients. An open-label, randomized comparison of talacotuzumab in combination with decitabine versus decitabine alone was also investigated. The recommended phase 2 dose was 9mg/kg. CR was achieved in 15% of patients receiving combination therapy versus 11% in those receiving decitabine alone. Median OS was 5.36 months for combination therapy versus 7.26 months for decitabine alone. Therefore, combination therapy did not demonstrate improvement in efficacy versus decitabine alone.

### 4.4 CXCR4

C-X-C chemokine receptor type 4 (CXCR4) is an important receptor in homing hematopoietic cells to bone marrow and is expressed highly in many hematological malignancies, identifying another favorable immunotherapeutic target in AML. However, utilizing CXCR4 as a target does have some challenges as it is also relatively expressed on normal hematopoietic cells and other tissues. Current work on a CXCR4 ADC is all preclinical, but Costa et al. showed promising work empirically deriving optimal ADCs (56). They optimized ADCs by varying linker-payload cleavability, drug-to-antibody ratio, affinity, and Fc format to maintain a tolerable safety profile while targeting more broadly expressed proteins. Their ADC which targets CXCR4 using auristatin greatly increased survival in MV4-11 AML mouse models, among other cancers. They observed significant differences in efficacy between different constructs of the ADC. More work must be done to expand this to other ADCs and see how this translates into targeting CXCR4 in clinical settings (56).

### 4.5 FLT3

Fms-like tyrosine kinase 3 (FLT3) is a class III receptor tyrosine kinase expressed on leukemic stem cells and blast cells of most AML patients with significantly higher expression levels than healthy tissue. Additionally, the presence of internal tandem duplication (ITD) mutation of FLT3 (FLT3-ITD) has a reportedly higher relapse risk and poorer outcome in AML patients (57). The 2023 Roas M. et al. preclinical study investigated 20D9-ADC (an ADC targeting FLT3) *in vivo* and *in vitro* models. *In vitro*, 20D9-ADC mediated potent cytotoxicity to *FLT3* or *FLT3-*ITD expressing Ba/F3 cells, AML cell lines, and *FLT3*-ITD positive patient-derived xenograft AML cells (**Table 2**). It also showed no severe hematotoxicity *in vitro* colony formation assays when using concentrations cytotoxic in AML cell line treatment. Additionally, 20D9-ADC treatment *in vivo* resulted in significant tumor reduction and complete remission in AML xenograft models (57).

## 5 Bispecific T-cell engagers

Bispecific T-cell Engager (BiTE) molecules function by having a single molecule interact with CD3 on T-cells and an antigen expressed by cancer cells. Like CAR-T therapies, this linkage directs a patient’s T cells to eliminate leukemic cells (58). The ideal targets for BiTE therapy are surface antigens that are selectively expressed on leukemic cells and have limited expression on normal cells. A key advantage over CAR-T is the “off-the-shelf” potential of these drugs. Unlike CAR-T, they are not processed from patient samples and can be given immediately. Blinatumomab is a CD19xCD3 bispecific BiTE molecule that has shown success in treating relapsed/refractory acute lymphoblastic leukemia. The success of blinatumomab has led to further investigation into alternative T-cell recruiting strategies in AML. Common treatment-related adverse effects include neurotoxicity, cytokine release syndrome, cytopenia, and hepatotoxicity (59).

### 5.1 FLT3 BiTE

FLT3 mutations are observed in around 25% of adult AML patients and 30% in those over 55 and are generally associated with poor outcomes (60). FLT3 also has limited expression in normal tissue, primarily found in early hematopoietic progenitors and leukemia blasts. Given this, FLT3 has been a promising target for several therapeutic options, including tyrosine kinase inhibitors and BiTEs (61). Two FLT3 BiTE molecules, one with a half-life extending moiety and one without, were analyzed by Brauchle et al. in 2020. They evaluated bulk leukemia cells from bone marrow or peripheral blood from 318 newly diagnosed or relapsed AML patients and found that 78% showed increased FLT3 protein expression. From there, they exposed FLT3 positive cell lines, such as MOLM-13, EOL-1, and MV4-11, to the BiTEs leading to cytotoxicity, whereas FLT3 negative cell lines, such as K562, showed relatively little response. Mouse xenograft models created using MOLM-13 and EOL-1 cells followed by injection of human CD3+ T cells showed a significant reduction in tumor growth with weekly FLT3 BiTE treatment. They also showed a reduction of FLT3 expression in cynomolgus monkeys with reasonable pharmacokinetics. The addition of PD-1 blockade also showed enhancement of cytotoxicity. This has been noted in other BiTE trials and is thought to be due to increased activation of T-cells inducing PD-1 expression (58).

### 5.2 CD123 BiTE

APVO436-5001 (NCT03647800) is an ongoing phase 1b dose escalation trial looking at APVO436, a novel bispecific anti-CD123 and anti-CD3 ADAPTIR molecule. The bispecific antibody has shown anti-leukemia activity in murine xenograft models of AML (62). Nine patients with R/R AML were treated in the study; and one patient achieved a prolonged stable disease, and two had a partial response that later progressed to a complete response (62). The most common grade 3 or greater adverse events likely APVO436-related were grade 3–4 cytokine release syndrome occurring in 4 of 46 patients (8.7%), grade 3–4 anemia occurring in 2 of 46 patients (4.3%), and infusion-related reaction occurring in 2 of 26 patients (4.3%). Further evaluation of this compound is ongoing.

Flotetuzumab (MGD006) is a bispecific antibody-based molecule to CD3 and CD123 in DART format. Preclinical models have investigated flotetuzumab mediated target-effector cell association, T-cell activation and proliferation, and potent killing in AML blasts both *in vitro* and *in vivo*. A phase 1/2 clinical study of flotetuzumab in R/R AML(NCT02152956) showed that of primary induction failure patients and early relapse patients, 26.7% achieved complete remission or complete remission with partial hematologic recovery. ORR was 30.0% with a median OS of 10.2 months, a 6-month survival rate of 75%, and a 12-month survival rate 50% (63). Unfortunately, due to underwhelming responses, the development of this product was discontinued (64).

### 5.3 CD123-FLT3 BiTE

One mechanism of resistance to BiTE therapy has been a loss of tumor-associated antigens. It was hypothesized that targeting more than one tumor-associated antigen could lead to a reduced frequency of relapse (65). The study used a half-life extended CD123-FLT3 BiTE molecule *in vitro*, mouse xenografts, and non-human primate tolerability studies. A survival benefit of >3 weeks was seen in the xenograft model (65). Additionally, both arms of the molecule were active, as survival benefits in mice with single positive tumors could not be calculated due to the high survival. Furthermore, follow-up blood tests showed a decrease in FLT3 mRNA. Repeat dosing was also not tolerated, and cytokine release was observed. It is hypothesized that CD123 expression would be increased with the cytokine release following repeat dosing.

### 5.4 CD33 BiTE

CD33 is expressed in >99% of AML cases, and BiTE has been effective in relapsed/refractory acute lymphoblastic leukemia. AMG 330 is a BiTE that binds to CD33 and CD3 on T cells and helps cause T-cell mediated destruction of CD33+ cells. In the presence of AML cells, AMG 330 induced the release of IFN-y, TNF, IL-2, IL-10, and IL-6 (66).

Ravandi et al. treated 35 relapsed/refractory AML patients with 12 different dose levels of AMG 330 ranging from 0.5-480 μg/d in a phase 1 study (**Table 3**). Of the thirty-five patients treated, thirty-one ultimately discontinued treatment. The majority of patients stopped therapy due to disease progression (n=24). Five patients discontinued therapy due to adverse events (two treatment-related), and two at the patient’s request. Overall, 23 adverse events occurred, 15 of which were treatment related. Most commonly, cytokine release syndrome (n=11), febrile neutropenia (n=6), pneumonia (n=4), leukopenia (n=3), and thrombocytopenia (n=2). Five patients demonstrated a response: two patients achieved CR, two achieved CRi, and had a morphological leukemia-free state (67).

## 6 Adoptive cell therapy

### 6.1 CAR-T-cell therapy

CAR-T is of great interest in AML. However, there remains a need for identifiable targets ubiquitously expressed in AML yet dispensable (**Table 4**) (68).

**Table 4:** Trials and studies using vaccine therapy.

Trial Identifier	Study Name	Status	Study Design	Phase	Study Drug	Drug Class	Disease setting/Study population	Results	Safety
NCT00965224	Efficacy of Dendritic Cell Therapy for Myeloid Leukemia and Myeloma			II		WT1	AML at high risk of relapse	13/30 patients had an antileukemic response with 9 achieving molecular remission, 5 of which were sustained after a median follow-up of 109.4 months, and 4 others showed disease stabilization. Five-year overall survival (OS) was higher in responders than in nonresponders (53.8% vs 25.0%; P = .01). In patients receiving DCs in first complete remission (CR1), there was a vaccine-induced relapse reduction rate of 25%, and 5-year relapse-free survival was higher in responders than in nonresponders (50% vs 7.7%; P < .0001).	Not reported
NCT01734304	Toll‐like receptor 7/8‐matured RNA‐transduced dendritic cells as post‐remission therapy in acute myeloid leukaemia: results of a phase I trial			I		WT1 and PRAME	AML in remission	No stastically significant difference between responders and nonresponders	Vaccination was well tolerated with only one grade 3 pyrexia. Other grade 1 and 2 reactioins included injection site reactions, musculoskeletal pain, other skin reactions, diarrhea and fatigue,
NCT02405338	WT1 and PRAME RNA-loaded dendritic cell vaccine as maintenance therapy in *de novo* AML after intensive induction chemotherapy			I/II		WT1 and PRAME	AML in remission	75% 5-year OS	Well tolerated with most AEs being grade 1 or 2 most commonly injection site reactions. 2 patients expereince grade 3 toxicity ( 1 herpes zoster and 1 upper respiratory infection)
NCT01373515	Leukemic Dendritic Cell Vaccination in Patients With Acute Myeloid Leukemia			I/II		WT1 and PRAME	AML at high risk of relapse	Vaccination with DC vaccine DCP-001 was safe and generated celular and humoral responses in 7/12 patients. Follow-up data showed increased survival in responding patients.	No significant AEs. Primarily injection site reactions.
NCT03697707	Efficacy and Safety of Immunotherapy With Allogeneic Dendritic Cells, DCP-001, in Patients With Acute Myeloid Leukaemia (ADVANCE-II)			II		WT1 and PRAME	AML at high risk of relapse	Preliminary results from 20 patients reported at ASH 2023. They report findings suggesting vaccination may increase dendritic cell populations leading to increase T cell activity against tumors. 2-year RFS and OS was estimated at 59% and 73% respectively.	No grade 3 or higher AEs related to the treatment have been reported. Related AEs mainly injection site reactions.
NCT01834248	NY-ESO-1 Vaccination in Combination with Decitabine Induces Antigen-Specific T-Lymphocyte Responses in Patients with Myelodysplastic Syndrome			I			MDS	Out of 9, a total of 7 patients completed the entire treatment course, two discontinued study treatment due to events deemed unrelated to the vaccine. All 7 patients showed NY-ESO-1 expression induction. Of those 7, 6 showed NY-ESO-1 specific CD4+ cell responses and 4 showed NY-ESO-1 specific CD8+ cell responses	The most frequent adverse events were deemed related to decitabine or the underlying hematologic malignancy and included cytopenias (predominantly grades 3/4), elevated liver enzymes (grade 3), fatigue (grade 2), edema (grade 2/3), and diarrhea.
NCT01956630	Clinical Study of DC Plus CIK for Patients With Relapse Acute Leukemia After Allo-HSCT			I		Survivin and MUC1	Relapsed AML	Increase in the 3-year probability of overall survival was seen, 48.9% in the intervention group vs 27.5% in control, Phase 2 look at 12 AML patients with 10/12 achieving remission	Overall, no grade 3 or grade 4 graft-versus-host disease was seen. Although other AEs were not generally commented on.

#### 6.1.1 CD123

There is a current ongoing trial (NCT02159495) where patients are being administered autologous anti-CD123 CAR-T cells for patients with CD123+ relapsed or refractory AML or CD123+ persistent or recurrent Blastic Plasmacytoid Dendritic Cell Neoplasm (69). The CD123CAR contains an anti-CD123 single-chain variable fragment, an optimized IgG4 CH2CH3 linker, a CD28 costimulatory domain, and a CD3 zeta signaling domain. The AML cohort contains six patients who have refractory AML after allogeneic HSCT and between 4-7 prior lines of therapy. Of two patients who received dose level 1, one achieved a morphologic leukemic-free state for two months. After the second infusion, blasts were reduced from 77.9% to 0.9%. Four patients received dose level 2. One of the four achieved a complete response and was transfusion-independent. The patient received another allogeneic HSCT and has remained in measurable residual disease (MRD)-negative complete response. Another patient also achieved a complete response. The other two patients did not achieve remission; however, they did have blast reductions.

Regarding safety, all toxicities were reversible. Four had grade 1 cytokine release syndrome (CRS), and one had grade 2 CRS. One developed adenoviral pneumonia, necessitating intubation. Lastly, one developed a grade 3 hypersensitivity rash. There were no dose-limiting toxicities (DLTs) and no treatment-related cytopenias. One patient with prior allogeneic HSCT developed cutaneous GVHD that was mild and recurrent after CAR-T therapy (69).

Another clinical trial of NCT03190278 is a phase I open-label trial looking at the safety and efficacy of off-the-shelf allogeneic UCART123v1.2 targeting CD123 in relapsed/refractory AML (70). There were two lymphodepletion regimen arms: one receiving fludarabine and cyclophosphamide (FC), while the other arm received fludarabine, cyclophosphamide, and alemtuzumab (FCA) to target CD52 in an attempt to reduce GVHD. Of 17 patients, four demonstrated clinical benefit. One FCA patient had greater than 90% blast reduction from 60% to 5% at day 28 post-infusion. Another FCA patient achieved a long-term MRD-negative complete response at 56 days post-infusion, which persisted for more than 12 months. The two other patients who achieved clinical benefit were part of the FC arm. One achieved blast clearance after day 14 post-infusion; however, by day 28 post-infusion, one had a rapid return of blasts. The other patient achieved a morphologic leukemic-free state. Overall, the FCA arm achieved prolonged lymphodepletion and UCART expansion compared to the FC arm. Accrual for this trial is ongoing.

#### 6.1.2 CD33

NCT03126864 is a single-center, single-arm phase I trial that looked at the tolerability of 3 different doses of the autologous CD33-CAR-T cells in relapsed/refractory CD33-positive AML (71). These cells were modified to express a CD33-targeted CAR with 4-1BB and CD3ζ endo-domains, which were co-expressed with truncated human epidermal growth factor receptor (HER1t). Ten adults were enrolled, where patients received a median of 5 prior treatments, and 3 were post-allogeneic stem cell transplant patients. One patient died before receiving CAR-T. There were no DLTs at dose level 1 during the 28-day DLT assessment period. Two patients developed CRS; one patient developed immune effector cell-associated neurotoxicity syndrome (ICANS). Adverse effects from CAR-T for one patient include grade 3 tumor lysis syndrome with acute kidney injury, grade 2 mucositis, and grade 1 tachycardia. Another patient developed grade 2 orthostatic hypotension, grade 2 increased bilirubin, and grade 3 transaminitis. One patient did not experience any toxicity. All three patients who received this CAR-T therapy died due to disease progression. Despite detectable anti-CD-33 CAR-T in blood samples, no anti-leukemic responses were identified (71). The authors state that due to challenges, including the aggressive nature of relapsed/refractory AML and the need for expeditious autologous product generation, logistic, and enrollment challenges, the trial was closed to patient entry following enrollment of the eleventh patient. The sponsors have transitioned to a platform that facilitates more rapid production and *in vivo* expansion with a product called PRGN-3006 (NCT03927261) (72, 73).

Many of the developed CAR-T target antigens are also expressed on normal cells, which can lead to off target toxicity (74). Guo and colleagues the expression profiles of CAR-T target antigens (CD33, IL3RA, and CLEC12A) at a single-cell level, and found significant upregulation of CD33 and IL3RA compared to normal BM HSPC and CLEC12A was strongly positive in almost all AML samples (74). However, these expression levels were highly variable during treatment. Therefore, in the development of this modality of therapy it is critical for the therapeutic targets to have minimal expression on healthy tissue to avoid unnecessary adverse effects.

#### 6.1.3 CD38

CD38 is an overexpressed tumor antigen in AML. Glisovec-Aplenc et al. developed autologous CD38-CAR T cells with 41BB-CD3ζ costimulatory domain in 2023 *in vitro* study (75). CD38 expression on activated T cells did not diminish CART-38 cell expansion or *in vitro* function. In xenografted mice, CART-38 rejection of AML cell lines was observed, and the overall survival of the animals was prolonged in this model. A decrease in hematologic progenitors was observed in a xenograft model of normal human hematopoiesis, which may pose a risk of decreased hematopoietic cell lines in patients in clinical trials (75). In a phase I/II (NCT04351022) clinical trial, CD38-directed CAR-T is designed to target CD38-positive R/R AML. Of the 6 patients treated in the study, two achieved CR or CR with incomplete count recovery (CRi) one week after CAR-T infusion. Four patients achieved CR or CRi 2 weeks and four weeks after CAR-T infusion. The 6-month OS rate and median OS was 50% and 12.3 months respectively. The leukemia-free survival rate and median leukemia-free survival were 50% and 10.3 months, respectively. Five patients developed grade I-II CRS and one developed grade III hepatotoxicity. No neurological toxicities were observed, but all six patients developed grade 3/4 hematological toxicities (76).

#### 6.1.4 CD7

CD7 CAR-T therapy has shown significant efficacy with a tolerable safety profile in T-cell lymphoid malignancies. A phase I clinical study (NCT04938115) investigates the efficacy and safety of CD7 CAR-T (NS7CAR-T) therapy in CD7-positive R/R AML patients. After four weeks of NS7CAR-T cell infusion, 7 of 10 patients (70%) achieved CR in BM. Six of these patients achieved MRD-negative CR. Three patients showed no remission. Of the 7 patients who achieved CR, 3 patients who relapsed from prior transplants did undergo a 2nd allogeneic HSCT two months after NS7CAR-T therapy. Two of the three remained leukemia-free on day 752 and day 315, respectively. However, the remaining patient died on day 241 due to transplant-related mortality. Regarding safety, 80% of patients had mild CRS, 7 patients had grade 1 CRS, one had grade 2 CRS, and two patients had grade 3 CRS. None of the patients had neurotoxicity. One of the patients with prior allogeneic HSCT, developed mild skin GVHD after CAR-T therapy (77).

#### 6.1.5 CLL-1

CLL-1 is a receptor highly expressed in AML cells. However, it is not found in healthy HSCs. The caveat is that CLL-1 expression is observed on all monocytes, early hematopoietic cells, and rarely non-hematological cells (78). In the 2022 Jin et al. phase 1 clinical study, 70% of R/R AML patients with CLL-1 CAR-T cells observed complete response with incomplete hematologic recovery (79). The median follow-up time was 173 days. All patients developed cytokine release syndrome (four of which were low-grade, six being high-grade). No patient developed CAR-T cell-related encephalopathy syndrome. All patients experienced severe pancytopenia, and two died due to infection from chronic agranulocytosis due to off-target toxicity.

#### 6.1.6 NKG2DL

NKG2D ligands (NKG2DL) are typically upregulated in cells that undergo malignant transformation, and other settings and are typically absent on healthy tissue (80). Additionally, NKG2DL has been reported to be expressed on AML cells and is an attractive target (80). In a phase I trial, NKG2DL was evaluated as a target for autologous CAR-T cells, with one patient experiencing a transient response (81). The THINK study is a multinational (EU/US) open-label Phase I trial that looked at CYAD-01, which is a CAR engineered with NKG2D receptor (82). The population studied is patients with R/R AML, myelodysplastic syndromes, or multiple myeloma with at least one line of treatment therapy. These patients are based in five hospitals in the USA and Belgium. Regarding efficacy, 25% achieved an objective response. Of the AML patients, three were treated with dose level 1 of CYAD-01, three were treated with dose level 2, and six were treated with dose level 3. Of the 16 patients treated, 44% had grade 3 or 4 TRAEs, and 31% had grade 3 or 4 CRS. One DLT of CRS was observed with dose level 3. No treatment-related deaths were observed.

### 6.2 TCR-T therapy

Similar to CAR-T cells, TCR-T cells are engineered T cells. However, instead of a synthetic receptor (CAR), they express a naturally occurring T cell receptor (TCR) that targets specific tumor antigens. The targeted antigens must be highly expressed on leukemic cells but limited to no expression on healthy hematopoietic cells. If the chosen antigen is found in healthy cells, TCR-T has to be short-lived. Several tumor-associated antigens (TAA) have been identified for preclinical trials with TCR-T. These include categories: overexpressed antigens (i.e. surviving, TERT), lineage-restricted antigens (i.e., WT1), minor histocompatibility A (HA)-1, neoantigens (i.e., NPM1 and CBFB-MHY11), and cancer-testis antigen (i.e., NY-ESO-1, MAGE, PRAME) (83).

A 2017 preclinical study showed that TERT TCR-T efficiently lyses AML cells *in vitro* and inhibits tumor growth in the AML xenograft model (84). A 2018 preclinical study showed that HA1 TCR-T demonstrated potential killing in AML/LCL cell lines (85). A 2020 preclinical study using CBFB-MHY11 for TCR-T exhibited potent antileukemic activity against AML cells and in the xenograft model (86). Lastly, a 2020 preclinical study with HLA-DPB1 TCR demonstrated highly lysed AML *in vitro* and in xenograft model (87). Most recent clinical trials utilize WT1 as the TAA. A phase I clinical trial (NCT01640301) used EBV-specific donor CD8+ T cells inserted with WT1-specific TCR amongst 12 AML patients with high risk for HCT-related relapse. High-risk patients were identified by genetics, including HLA-A*0201 expression. Relapse-free survival was achieved in 100% of patients with a median of 44 months after infusion (88). Regarding safety, two patients developed grade III CRS. Eight patients developed grade III lymphopenia, and five patients developed grade IV lymphopenia. Two patients developed grade III thrombocytopenia, one exhibited grade III reduced neutrophil count, and one exhibited grade IV reduced neutrophil count. Lastly, seven patients developed grade III anemia.

### 6.3 Tumor-infiltrating lymphocytes

Tumor-infiltrating lymphocytes infiltrate the tumor microenvironment and have the potential to recognize and kill cancer cells (89). One study investigated ex vivo isolation, expansion, and bioengineering of specific subsets of tumor-infiltrating lymphocytes (TILs) from the bone marrow of AML patients (90). Two subsets of TILS CCR7+CD95-/or and CD62L+CD45RA+ have been shown to possess anti-tumor activity (90). Researchers were able to isolate the desired TILS through the use of magnetic-activated cell sorting and flow cytometry.

Novel approaches are being analyzed to target AML while sparing normal hematopoiesis. Researchers also used bioengineering of TILs to enhance anti-tumor activity by using gene-edited hematopoietic stem and progenitor cells (HSPCs) to regenerate an antigen-negative myeloid system (91). They genetically modified TILs to express a chimeric antigen receptor (CAR) that targeted CD33, which is highly expressed in AML. The study found that *in vitro* differentiated CD33 KO HSPCs had reduced levels of CD33 protein expression (27% ± 4%) compared to controls (92% ± 3%) (91). Mice were injected with control, or CD33 KO HSPCs, and their human cell engraftment was followed over time in peripheral blood. It was found that mice who were injected with CD33 KO HSPC had diminished expression of CD33 sustained over time. The total number of CD45+ cells and myeloid subsets remained nearly identical between the two groups. The CAR-modified TILS showed enhanced cytotoxicity against AML cells *in vitro*, which suggests the potential ability to improve the efficacy of TIL-based immunotherapy in AML.

TIL-based treatments for AML are in the early stages of development with multiple possible beneficial combinational therapeutic approaches. TILs combined with hypomethylating agents and checkpoint inhibitors may enhance their anti-tumor activity.

### 6.4 NK cell therapy

Natural killer (NK) cells play an enormous role in innate immunity by targeting infectious agents and tumor cells. The interplay between NK cells and cytokine secretion is dictated by activating or inhibitory receptors that bind ligands on these target cells, ultimately allowing NK cell activation and killing targets (92). In AML, malignant cells evade the immune system by suppressing NK cell function. NK cell therapy is a novel immunologic approach to treatment in AML, in which suppression is prevented, allowing these cells to perform cell-specific targeted killing.

There have been challenges with harvesting NK cell lines for therapy. NK cells from donor peripheral blood mononuclear cells (PBMCs) express markers like CD16, NKG2D, NKp44, and NKp46, which allow them to recognize and kill non-self-cells such as tumor cells. Unfortunately, the quantity of NK cells in peripheral blood is low and not cost- or time-effective. Induced pluripotent stem cells are a good source of NK cells. However, they express low levels of CD16, therefore making them less cytotoxic. NK-92 cell lines are an alternative option. They are immortalized NK lymphoma cells; but have their own safety concerns (92).

CD94-NKG2A is another target for immune checkpoint therapy. It is an inhibitory receptor on NK cells and CD8+ T cells that inhibits the cytotoxic function of these respective cells (93). This receptor is inhibited by HLA-E, a non-classical MHC class I molecule found on normal and neoplastic hematopoietic cells. The 2016 Ruggeri, L. et al. study demonstrated that the humanized anti-NKG2A antibody converts NKG2A+ NK cells into effector NK cells that induce tumor cell death by killing most HLA-E+ NK resistant lymphohematopoietic cells such as dendritic cells, myeloid cells, B and T lymphocytes, and leukemic cells including AML amongst other cell types.

Recently, interest in memory NK cells has grown. These cells have differentiated in response to IL-12, IL-15, and IL-18 cytokines. These cells are specifically called cytokine-induced memory-like differentiation (ML NK) and have shown enhanced effector function and prolonged survival in various leukemic models (94). A phase I/II clinical trial investigates the best dose and side effects of activated natural NK cells in treating patients with R/R AML and R/R MDS. Chemotherapy will be given before donor NK cell infusions, which will slow the growth of cancer cells and prevent the recipient’s immune system from rejecting these donor cells. NK infusion will be from a related MHC-haploidentical donor. These modified NK cells may help strengthen the immune response to targeting and killing leukemic cells. Aldesleukin, an IL-2 cytokine, will be administered for two weeks following NK infusion to support NK growth. The study had 15 patients, 7 of whom achieved complete remission, supporting the use of allogeneic ML NK cells as therapy for relapsed AML. The caveat is that allogeneic donor NK cells were eliminated in several weeks after receiving T-cell recovery. Therefore, ML NK cell persistence could not be investigated (94).

A phase II clinical trial does study ML NK cell persistence. Patients with relapsed AML who received haploidentical hematopoietic cell transplants also received ML NK cells from their HCT donors, preventing NK cell allo-rejection. On day 28, complete remission was achieved in more than 90% of patients. Patients who have relapsed with AML after HCT and who received salvage therapy followed by T cell donor lymphocyte infusion (DLI) showed poorer outcomes. Therefore, the effects of donor ML NK infusion added to salvage therapy and T cell DLI for relapsed AML patients after HSCT were studied. The salvage therapy was fludarabine, cytarabine, and granulocyte-colony stimulating factor. T cell DLI and ML NK infusions were derived from the same HCT donor. The NK infusions were safe, and no patient experienced cytokine release syndrome. This regimen was well tolerated; only one patient exhibited mild skin GVHD. Within one month, 4 of 9 patients achieved complete remission, and 2 achieved partial remission. On day 100, two more patients reached complete remission. Additionally, with this immune-compatible therapy, ML NK cells persisted in PB and bone marrow for at least three months in most patients. These ML NK cells drawn from recipient PB, or bone marrow displayed anti-leukemic characteristics within *in vitro* assays and retained their phenotype over time (95).

### 6.5 Pluripotent stem cell-derived CAR-T and TCR-T therapy

Edited T cells are a significant area of study for CAR-T Therapy, however, this approach faces several limitations, including the availability of autologous T cells, complications with therapies, and resistance (96–98). The tumor killing ability of the T cells is also suppressed by increasing Treg subsets and senescent and exhausted T compartment (9, 99).

Research has demonstrated that mouse OP9 stromal cells expressing Notch logan DLL1 can induce T cell lineage commitment *in vitro* from natural mouse hematopoietic progenitor cells (100). This was later expanded with the use of human blood progenitor cells in the presence of OP9 stromal cells expressing human NOTCH1 ligand DLL1 (101). This work has illustrated the ability to generate T-cells from hematopoietic stem cells utilizing an *in vitro* co-culture system.

One of the future goals is to develop a method capable of generating immunocompetent and therapeutic T lymphopoiesis from unlimited and gene-editable PSCs (102). Pluripotent stem cells have a unique advantage, with efficient gene editing and self-renewal properties *in vitro*, making them valuable candidates for T-cell generation (103). Runx1 and Hoxa9, during the early stages of endothelial-to-hematopoietic transition to hematopoietic progenitor maturation in the PSC differentiation scheme *in vitro*, produced a type of induced hematopoietic progenitor cells (iHPCs) that could home to the thymus, engraft, and generate induced T cells (iT cells) with diverse TCRαβ repertoire in immunodeficient mice (102). These iT cells helped restore immune surveillance function in immunodeficient mice and also possessed anti-tumor activities *in vivo* when engineered to carry tumor antigen-specific TCR at the PSC stage. These findings help create a potential link to the unlimited and editable PSC source and T cell-based immunotherapy, potentially overcoming the current limitations faced by CAR-T therapies.

## 7 Vaccine therapy

There is a growing focus on antileukemic vaccinations as therapy for active leukemia and prevention of relapse in high-risk patients (**Table 5**). Vaccine therapies are designed to elicit cellular and humoral immune responses against leukemic myeloid blasts by targeting overexpressed or specific leukemic antigens, i.e., Tumor-associated antigens (TAAs). Current TAAs targets include Wilms tumor 1 (WT1), Preferentially expressed antigen of melanoma (PRAME), Proteinase 3 (PR3), receptor for hyaluronic acid-mediated motility (RHAMM), New York esophageal squamous cell carcinoma 1 (NY-ESO-1) and mucin 1 protein (MUC1) with WT1 being the most actively studied at this time (104). While conventional peptide-based vaccines are being studied, current research on eliciting dendritic cells as key antigen-presenting cells also shows promise. Dendritic cell dysfunction is an essential method of evasion in leukemia progression. However, by taking dendritic cells and loading them with these TAAs, there is hope to close this gap in immune cancer surveillance. This approach remains early in development in AML and may require a combinatorial approach that includes agents that augment the adaptive immune system. These trials have generally shown tolerable safety profiles, with many reporting only injection site irritation. Larger phase II/III trials will be required to determine the true potential of these therapies.

**Table 5:** Preclinical Studies investigating several upcoming therapy targets.

Drug Class	Drug/Therapy	Enrollment	Disease setting/Study Population	Results
TIGIT		38 PB and 32 BM samples from *de novo* AML patients; 17 PB and 21 BM samples from CR AML; 10 PB and 8 BM samples from R/R AML; 36 PB samples from healthy donors and 14 BM samples from healthy donors/IDA donors	*De novo* AML, R/R AML	Significantly higher co-expression of TIGIT and PD-1 in BM samples of *de novo* AML patients compared to PB samples
TIGIT			Newly diagnosed and relapsed AML	TIGIT highly expressed on BM-infiltrating immunosuppresive M2 macrophages
TIGIT		37 PB samples from AML/MDS patients in remission; 20 PB samples from healthy donors	Post-allogeneic HSCT AML/MDS in remission	High levels of TIGIT on CD4+ T cells 3 months after allogeneic HSCT was significantly associated with an increased risk of relapse. Higher expression levels of TIGIT on CD4+ T cells was significantly correlated to poorer clinical outcomes
LAG3				LAG3 expression on CD8+ T cells co-cultured with irradiated K1: 11.8% ± 2.4 v. PBMC co-cultured with non-irradiated K1 cells: 17.5% ± 2.5; n=4, P=0.002. Anti-LAG3 antibody in PBMC co-cultured with non-irradiated AML cells: 16.3% IFNg on CD8+ T cells, 6.5% CD137 on CD8+ T cells, 1.7% Tregs. Without anti-LAG3 antibody: 6.6 % IFNg on CD8+ T cells, 4.1% CD137 on CD8+ T cells, Tregs 3.8% (IFNg: P=0.01, n=4; CD137: p=.007, n=4; Tregs: P=.04, n=4)
LAG3		176 patient data, 62 *de novo* AML BM samples	AML	AML patients have poorer overall survival with high CTLA-4 and LAG3 expression (3-year OS 9% vs 36% and 13% vs 40% respectively).
TIM3		40 samples from 15 AML patients, and 7 healthy donor controls	AML	TIM3 is upregulated in cell lysates of patients with AML as compared with healthy controls.
Anti-CD123	mPO-6	n/a	Refractory AML mouse model	CD123 antagonistic peptide micelle formulation mPO-6 can significantly enhance apoptosis and prolong the survival of AML mice by effectively interfering with the axis of CD123/IL-3
Anti-CD123	SIRPα-αCD123	n/a	AML	SIRPα-αCD123 fusion antibodies disrupted CD47/SIRPα signalling in AML cell lines and specifically enhanced leukemia stem cell clearance.
TERT	hTERT865-873-specific, TCR-engineered T-cells	10 AML, and 10 B-ALL patient samples with healthy donor comparisons	AML/ALL	T cells transduced with an HLA-A2-restricted T-cell receptor (TCR), which recognize human TERT with high avidity, lysed AML and ALL cells *in vitro* and limited progression in AML and B-ALL xenograft models.
HA1	HA-1 TCR transgene contruct inserted into T cells	n/a	AML/ALL	HA-1 is a minor histocompatibility antigen that is highly expressed in leukemic cells and hematopoetic cells, but not non hematopoetic cells. CD4+ and CD8+ T cells transduced with a lentiviral vector incorporating an HA-1 TCR transgene construct were able to specfically kill HA-1 positive cells.
CBFB-MHY11	CBFB-MYH11 epitope–specific T cell receptor transduced T cells	n/a	AML	Highly-avid healthy donor CD8+ T cell clones killed CBFB-MYH11+ HLA-B*40:01+ AML cell lines and primary human AML samples *in vitro*. CBFB-MYH11–specific T cells also controlled CBFB-MYH11+ HLA-B*40:01+ AML in vivo in a patient-derived murine xenograft model. Transduction of high-avidity CBFB-MYH11 epitope–specific T cell receptor into CD8+ T cells conferred antileukemic potential.
HLA-DPB1	HLA-DPB1 specific T cell receptor transduced CD4 and CD8 T cells	n/a	AML	HLA-DPB1 specific T cell receptor transduced CD4 and CD8 T cells showed strong reactivity to AML *in vitro*. CD4 cells were able to eliminate leukemia blasts in AML engrafted mice. However, strong cross-reactivity with non-hematopoietic cells is a concern and safety mechanisms will have to be investigated.
FLT3	Two novel FLT3 BiTE molecules, one with a half-life extending (HLE) Fc moiety and one without	16 AML patient samples	AML	FLT3 BiTE molecules induced T-cell dependent cytotoxicity of FLT3-positive cells *in vitro*, reduced tumor growth and increased survival in AML mouse models. Both molecules studied exhibited reproducible pharmacokinetic and pharmacodynamic profiles in non-human primates.
CD123-FLT3	half-life extended (HLE) CD123-FLT3 dBiTE 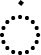 molecule	n/a	AML	CD123-FLT3 HLE dBiTE 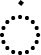 molecule was active against both double positive and single positive target cells *in vitro* and *in vivo*. However, some safety concerns were present with repeat dosing not being tolerated with cytokien release.
CD123 BAT & CD33GO BAT	CD123 Bispecific antibody armed activated T cells (BATS) or CD33GO BATs	13 AML patient samples, 3 Healthy donors	AML	AML-engrafted NSG mice showed significantly prolonged survival in mice treated with CD33GO BATs (p < 0.0001) or CD123 BATs (p < 0.0089) compared to ATC-treated control mice. Patient samples containing leukemic blasts and LSCs when treated with CD33GO BATs or CD123 BATs for 18 h showed a significant reduction (50%–100%; p < 0.005) in blasts and 75%–100% reduction in LSCs (p < 0.005) in most cases compared to unarmed ATCs.
FLT3	20D9-ADC	n/a	AML	20D9-ADC was cytotoxicity to Ba/F3 cells expressing transgenic FLT3 or FLT3-ITD, to AML cell lines, and to FLT3-ITD–positive patient-derived xenograft AML cells. In vivo, 20D9-ADC treatment led to tumor burden reduction and even remission in AML xenograft models.
CD123 ADC	IMGN632	n/a	AML	Cytotoxicity against AML samples *in vitro* and in xenograft mouse models with less hematopoietic toxicity than previous ADCs thought to be due to using indolinobenzapine as cytotoxic drug over drugs like pyrrolobenzodiazepine
CXCR4	CXCR4 ADC	n/a	AML	ADC targeting CXCR4 using different linker-payload cleavability, drug-to-antibody ratio (DAR), affinity and Fc formats showed effiicacy and promising tolerabilty in MV4-11 mouse models.
NKG2A	Anti-human NKG2A antibody	n/a	Primary AML and EBV cell lines	Mice co-infused with human primary leukemia or Epstein-Barr virus cell lines and NKG2A+ natural killer cells, pre-treated with anti-human NKG2A, were rescued from disease progression.
DC Vaccine	anti-hDEC205-WT110–35, anti-hDEC205-WT191–138, anti-hDEC205-WT1223–273, anti-hDEC205-WT1324–371 vaccines	8 AML, 2 MDS/CMML post-HSCT samples and 6 healthy patients donors	AML/MDS	Anti-hDEC205-WT191–138 vaccine was capable of directly inducing ex vivo T-cell responses by co-incubation of the fusion protein-loaded monocyte-derived mature DCs and autologous T-cells of either healthy or HSCT individuals. Furthermore, the DC-targeted WT191–138-induced specific T-cells showed a strong cytotoxic activity by lysing WT1-overexpressing THP-1 leukemia cells *in vitro* while sparing WT1-negative hematopoietic cells.
IDO inhibitor	Indoximod	184 AML Patients	AML	IDO actiivty was increased in AML patient samples compared to healthy individuals.
Sting Agonist	GSK3745417	n/a	AML	GSK3745417 had an inhibitory effect on 11/13 AML cell lines. Caspase induciton was seen in 7 out of the 11 responding cell lines.
Sting Agonist	SHR1032	n/a	AML	Novel small molecule non-CDN STING agonist SHR1032 induced apoptosis in AMP cell lines

### 7.1 WT1

High WNT1 expression is associated with poor prognosis, which is often seen in AML. Anguille et al. reported from their phase II trial investigating dendritic cell vaccination. The study had 30 AML patients who were at increased risk for relapse were vaccinated with dendritic cells that were electroporated with WT1 mRNA. There were 13 of 30 study participants who responded to treatment, with 9 achieving molecular remission measures by normalization of WT1 levels and 4 showing disease stabilization. The five-year overall survival of the responders was 53.8% vs 25.0% in the nonresponder group (105). While this initial data is promising, larger control trials will need to be done, given that this study primarily compared nonresponders and responders with a relatively small sample size. Ogasawara et al. also studied 9 AML and 2 acute lymphoblastic leukemia (ALL) patients treated with peptide-loaded DCs in 2022. They saw an increase in progression-free survival, although no statistically significant increase in overall survival. They also observed a decrease in regulatory T cells following vaccination, suggesting DC vaccination may contribute to a reversal of immunosuppression.

Some groups have tried to increase the expression of these antigens on dendritic cells, with Dagvadori et al. creating a WTI-DEC205 fusion protein. This chimeric anti-hDEC205-WT1_91-138_ directly induced ex-vivo responses when fusion protein-loaded DC were co-incubated with T-cells. Additionally, the co-incubated T-cells showed a robust cytotoxic activity lysing WT1-overexpressing THP-1 leukemia cells while sparing WT1-negative hematopoietic cells *in vitro* (104).

### 7.2 WT1 and PRAME

Several other phase 1 trials have tried combining multiple TAAs with multiple trials giving vaccines with DCs loaded with WT1 and PRAME. Lichtenegger et al. examined 10 patients treated with vaccines using WT1 and PRAME. They saw a greater immune response in those under 65, but this did not reach a statistically significant difference between responder and nonresponder OS (106). Floisand, Y et al. looked at 20 patients who had a 5-year OS of 75% but did not directly compare this to a control or nonresponder group (106). Loosdrecht et al. looked at 12 patients treated with DCP-001, now known as vididencel, a dendritic vaccine using WT1 and PRAME. They observed an immune response in 7 of the 12 patients. Patients who responded had significantly increased OS (107, 108). A follow-up study, ADVANCE-II (NCT03697707) has preliminary results from 20 patients reported at ASH 2023. They reported findings suggesting vaccinations may increase dendritic cell populations, leading to increased T-cell activity against tumors; 2-year RFS and OS were estimated at 59% and 73%, respectively (109, 110).

### 7.3 NY-ESO-1

NY-ESO-1 has been a promising target in many cancers, given its expression profile in healthy tissue, mostly limited to embryonic development and germ cells. However, expression is seen in many cancers, and treatment with hypomethylating agents such as decitabine can enhance this expression by acting on the hypermethylated promoter of NY-ESO-1 (111). Griffith et al. looked to take advantage of this by combining decitabine with a NY-ESO-1 fusion protein vaccine in 9 MDS patients (112). A total of 7 patients completed the entire treatment course, and two discontinued study treatments due to events deemed unrelated to the vaccine. All 7 patients showed NY-ESO-1 expression induction. Of those 7, 6 showed NY-ESO-1 specific CD4+ cell responses, and 4 showed NY-ESO-1 specific CD8+ cell responses. Dendritic cells were again thought to be a key component in T-cell activation, with increased CD141^Hi^ DCs at diagnosis being associated with immune response (112).

### 7.4 Survivin and MUC1

NCT01956630 was a 2-stage, phase 1 trial that looked at using genetically modified DC carrying TAAs Survivin and MUC1 and an RNA interference moiety to suppress SOCS1. In Stage 1, safety was assessed, with 23 patients with relapsed AL receiving the intervention and 25 receiving standard donor lymphocytes. The treatment was well tolerated, with no documented stage 3 or 4 GVHD. An increase in the 3-year OS was seen 48.9% in the intervention group vs 27.5% in the control group. Stage 2 looked at 12 relapsed AML patients, with 83% achieving complete remission (113).

## 8 STING agonist therapy

Stimulator of Interferon Genes (STING) is a transmembrane protein in the endoplasmic reticulum first identified in 2008 as part of a signaling cascade leading to the production of type-1 interferon (IFN) among other inflammatory cytokines and signaling molecules (114). Type-1 IFN in sufficiently increased concentrations leads to cancer cell cycle arrest, modulation of apoptosis, and increased activity of tumor-invading lymphocytes (115). Endogenously activated by cytosolic DNA, this increased release of type-1 IFN is seen in solid tumors but is not seen in AML, suggesting a possible avenue to increase the host innate immune response to these malignancies (116).

Drugs targeting the pathway have mostly been cyclic di-nucleotide (CDN) mimics of endogenous ligands. However, these have had disappointing results thus far, with Novartis halting trials using its ADU-S100 compound due to a lack of efficacy in solid tumors. Newer small molecule agonists have generated interest with greater potency. Adam et al. demonstrated this with GSK3745417 exerting a growth inhibitory effect on 11 out of 13 tested AML cell lines (117). Further experiments showed caspase induction in 7 of those 11 cell lines, supporting an apoptotic mechanism behind the observed inhibition. The compound also blocked colony formation in AML samples from 5 donors (117). The makers, GlaxoSmithKline, are currently conducting a phase I trial to assess the therapeutic potential of this compound. Additionally, SHR1032, another non-CDN small molecule STING agonist, has generated interest in preclinical *in vivo* studies inducing apoptosis in AML (118).

## 9 IDO inhibitors

IDO (indoleamine 2,3-dioxygenase) is an enzyme that can suppress the immune system when activated. Inhibitors of this enzyme help counteract this suppression and allow the immune system to attack cancer cells effectively (119). Studies have shown that IDO is expressed by bone marrow and peripheral blood AML blasts, while CD34+ hematopoietic precursor cells do not express the IDO protein (119). Additionally, one study showed increased IDO enzymatic activity in the blood of AML patients compared to controls (120). One study also found that IDO and FOXP3 mRNA were upregulated in AML patients. There was a positive correlation between IDO and FOXP3, indicating that IDO expression may be associated with increased Treg phenotype (121). The inhibition of IDO may be a promising approach, along with chemotherapy, in patients with upregulated IDO expression.

Indoximod is an immunometabolic adjuvant that works through suppression of master metabolic kinase mTORC1 that occurs in tryptophan-depleted cells (122). The Indoximod method of action is essential because AML cells can often experience inhibition of mTORC1 activity, which can impair T cell function and immune response (122). One study analyzed IDO-1 expression by quantifying by a novel composite IDO-1 score’ on diagnostic bone marrow biopsies of AML patients. Univariate analysis of the study found higher IDO-1 mRNA (p=0.005), higher composite IDO-1 score (p<0.0001), and multivariate model retained higher composite IDO-1 score predicted poor survival outcomes. Patients who failed induction had a higher composite IDO-1 score (p=0.01).

Indoximod has been studied in clinical trials with idarubicin/cytarabine in newly diagnosed AML. The study was an open-label, multicenter, phase 1 study (NCT02835729) that analyzed newly diagnosed AML patients treated with indoximod in combination with induction chemotherapy. Overall, 31 patients were enrolled, with 25 completing the study. The most frequent AEs were febrile neutropenia (60%) and hypoxia (16%). Overall, 21/25 (84%) of patients achieved remission (CR/CRh/CRi/CRp), and 15 of 19 (79%) in the per-protocol analysis achieved remission. All patients in the study who received HiDAC became MRD-negative. The median relapsed free and overall survival have yet to be reported. The study aimed to enhance the efficacy of chemotherapy by targeting the IDO pathway and relieving tryptophan depletion-mediated mTORC1 suppression (123). However, currently, there are no studies to our knowledge that have reported data on IDO in relapsed/refractory AML.

## 10 Conclusions

Over the last several years, immunotherapy has revolutionized the treatment and outcomes of cancer patients. The role of immunotherapy in the treatment of patients with AML has come to light more clearly only recently. These studies evaluating various immunotherapeutic approaches remain in the very early stages with limited numbers of patients, although the results of these studies are encouraging with signals of efficacy.

This review summarizes the most up-to-date novel advances for immunotherapies in relapsed/refractory AML. It is of particular interest for those patients ineligible for allogeneic stem cell transplantation or with relapsed or refractory disease resistant to standard cytotoxic chemotherapies. However, barriers to the evolution of immunotherapy in AML include limited AML-specific antigen expression, which can result in off-target toxicity, as well as the suppressive and dysfunctional immune milieu in heavily pre-treated AML patients. Although there is much enthusiasm for the deployment of immunotherapy in AML, based on the data generated to date, it is clear that agents targeting only the innate vs. adaptive compartments are of limited value. In addition to chemotherapy, a combinatorial approach employing agents that target both the innate and adaptive may be warranted. Furthermore, the timing of administration of immunotherapy is likely crucial, as such approaches in the relapsed/refractory setting may be hindered by insufficient T-cells, exhausted T-cells, or inadequate antigen presenting cell function. Rational cellular therapeutics, in combination with some of the more recently approved targeted agents applied earlier in the disease course, may be of particular value.

Moreover, a better understanding of the immune microenvironment within AML can better inform the development of immunotherapeutic strategies for AML. Moreover, it is crucial to identify biomarkers of response to these immunotherapeutic agents to better identify patients that may benefit from these approaches.

In summary, the complexity of AML has led to the exploration of novel immunotherapies to help identify specific targets and minimize immune escape. Despite reservations regarding low antigenicity and having long been considered a “cold” tumor, immunotherapy remains a highly promising strategy for patients with AML.
